# Associations between body mass index and the risk of renal events in patients with type 2 diabetes

**DOI:** 10.1038/s41387-017-0012-y

**Published:** 2018-01-17

**Authors:** Kamel Mohammedi, John Chalmers, William Herrington, Qiang Li, Giuseppe Mancia, Michel Marre, Neil Poulter, Anthony Rodgers, Bryan Williams, Vlado Perkovic, Josef Coresh, Mark Woodward

**Affiliations:** 1 The George Institute for Global Health, University of Sydney, Sydney, Australia; 2 INSERM, UMRS 1138, Centre de Recherche des Cordeliers, Paris, France; 3 Department of Diabetology, Endocrinology and Nutrition, Assistance Publique Hôpitaux de Paris, Bichat Hospital, DHU FIRE, Paris, France; 4 Nuffield Department of Population Health, University of Oxford, Oxford, UK; 5 The University of Milan-Bicocca and Istituto Auxologico Italiano, Milan, Italy; 6 Sorbonne Paris Cité, UFR de Médecine, University Paris Diderot, Paris, France; 7 The International Centre for Circulatory Health, National Heart and Lung Institute, Imperial College, London, UK; 8 Institute of Cardiovascular Sciences, University College London (UCL) and NIHR UCL Hospitals Biomedical Research Centre, London, UK; 9 Department of Epidemiology, Johns Hopkins Bloomberg School of Public Health, Johns Hopkins University, Baltimore, MD USA; 10 The George Institute for Global Health, University of Oxford, Oxford, UK

## Abstract

**Background/objectives:**

We aimed to evaluate the relationship between BMI and the risk of renal disease in patients with type 2 diabetes in the Action in Diabetes and Vascular Disease: PreterAx and DiamicroN Modified-Release Controlled Evaluation (ADVANCE) study.

**Subjects/methods:**

Participants were divided into six baseline BMI categories: <18.5 (underweight, *n* = 58); ≥18.5 to <25 (normal, *n* = 2894); ≥25 to <30 (overweight, *n* = 4340); ≥30 to <35 (obesity grade 1, *n* = 2265); ≥35 to <40 (obesity grade 2, *n* = 744); and ≥40 kg/m^2^ (obesity grade 3, *n* = 294); those underweight were excluded. The composite outcome “major renal event” was defined as development of new macroalbuminuria, doubling of creatinine, end stage renal disease, or renal death. These outcomes and development of new microalbuminuria were considered individually as secondary endpoints.

**Results:**

During 5-years of follow-up, major renal events occurred in 487 (4.6%) patients. The risk increased with higher BMI. Multivariable-adjusted HRs (95% CIs), compared to normal weight, were: 0.91 (0.72–1.15) for overweight; 1.03 (0.77–1.37) for obesity grade 1; 1.42 (0.98–2.07) for grade 2; and 2.16 (1.34–3.48) for grade 3 (*p* for trend = 0.006). These findings were similar across subgroups by randomised interventions (intensive versus standard glucose control and perindopril-indapamide versus placebo). Every additional unit of BMI over 25 kg/m^2^ increased the risk of major renal events by 4 (1–6)%. Comparable results were observed with the risk of secondary endpoints.

**Conclusions:**

Higher BMI is an independent predictor of major renal events in patients with type 2 diabetes. Our findings encourage weight loss to improve nephroprotection in these patients.

## Introduction

Globally, obesity is common with alarming rates of increasing prevalence^1,2^. It is a key component of the metabolic syndrome, which is also characterised by hypertension, dyslipidaemia, and insulin resistance, and often leads to type 2 diabetes^3^. Diabetes is a leading cause of chronic kidney disease (CKD) and end-stage renal disease (ESRD)^4^. In the absence of diabetes, however, experimental and epidemiological studies have also provided accumulating evidence that obesity is an independent risk factor for CKD^5,6^, a risk mediated in part through intraglomerular hypertension and hyperfiltration^7^.

Most reported observational studies have found positive associations between being overweight or obese and kidney outcomes (which include development of CKD, rapid changes in kidney function or ESRD)^8–12^. However, few have been large enough to compare people with and without diabetes reliably^9^, and there remains some uncertainty in people with diabetes as to whether higher body mass index (BMI) increases risk of developing macroalbuminuria, and whether BMI–CKD associations are mediated through differences in renal risk factors affected by adiposity (e.g., glycemia and blood pressure).

In the present study, we aimed to evaluate the relationship between baseline BMI and major renal events among patients with type 2 diabetes in the Action in Diabetes and Vascular Disease: PreterAx and DiamicroN Modified-Release Controlled Evaluation (ADVANCE) trial (ClinicalTrials.gov number, NCT00145925). In order to investigate potential mechanisms for any associations, outcomes were analysed for the whole cohort and in subgroups for those randomised to different intensities of long-term glycaemic control and to use of placebo versus perindopril-indapamide, an ACE-inhibitor/diuretic combination drug that would be expected to reduce glomerular hyperfiltration.

## Materials/subjects and methods

### Study population

The ADVANCE study was a 2 × 2 factorial randomised controlled trial which tested the effects of intensive glucose control using a gliclazide-MR-based regimen, and routine blood pressure treatment using a fixed-dose combination of perindopril and indapamide, on the incidence of major macrovascular and microvascular events in patients with type 2 diabetes. The design and clinical characteristics of participants have been published previously^13–15^. Briefly, patients aged 55 years or older with diabetes diagnosed at 30 years or older with pre-existing cardiovascular disease or with at least one risk factor for cardiovascular disease were eligible. Participants were followed prospectively for clinical events and had blood pressure and urinary albumin to creatinine ratio (ACR) measured at local study clinics at 2-year, 4-year and final follow-up visits. The ADVANCE protocol was approved by the Institutional Ethics Committee of each participating centre and all participants provided written informed consent before their enrolment in the trial.

### Definition of BMI categories at baseline

Baseline BMI, computed as the weight in kilograms divided by the square of the height in metres, was categorised at baseline into six categories according to the World Health Organization classification^16^: underweight (<18.5), normal weight (≥18.5 to <25), overweight (≥25 to <30), and obesity grade 1 (≥30 to <35), grade 2 (≥35 to <40), and grade 3 (≥40 kg/m^2^).

### Primary and secondary endpoints

As pre-specified in the ADVANCE protocol^13^, “major renal events” were defined as a composite of macroalbuminuria (defined as a urinary ACR > 300 mg/g), doubling of the serum creatinine level to at least 200 μmol/l, ESRD (defined as the need for renal-replacement therapy), or death due to renal disease. “New cases of microalbuminuria” (defined as 30 < ACR ≤ 300 mg/g), “Development of new macroalbuminuria”, and “doubling of creatinine, ESRD, or renal death”, were considered individually as secondary endpoints. The primary endpoints were reviewed by an independent End Point Adjudication Committee.

### Statistical analyses

Clinical and biological characteristics of participants at baseline were presented both overall and according to BMI categories. Categorical variables were expressed as the number of patients with the corresponding percentage, and continuous variables as mean (SD), or as median (interquartile interval) for those with a skewed distribution. Patients with missing data regarding estimated glomerular filtration rate (eGFR) and ACR at baseline (*n* = 545) were excluded from the current study. Few (*n* = 58; 0.6 %) patients were underweight, and so these were also excluded from the main set of analyses, although included in a sensitivity analysis. Cox proportional hazards regression models were fitted to estimate hazard ratios (HRs), with associated 95% confidence intervals (CI), for major renal events by BMI categories, taking normal weight as the reference group. The primary model (model 1) adjusted for baseline age, sex, region of origin (Asia: Philippines, China, Malaysia, and India; established market economies: Australia, Canada, France, Germany, Ireland, Italy, Netherlands, New Zealand, United Kingdom; and Eastern Europe: Czech Republic, Estonia, Hungary, Lithuania, Poland, Russia, Slovakia), prior cardiovascular disease (defined as the presence at baseline of myocardial infarction, stroke, coronary artery bypass graft, percutaneous transluminal coronary angioplasty, hospital admission for unstable angina or transient ischaemic attack), eGFR (computed by the CKD–Epidemiology Collaboration equation)^17^, squared eGFR, urinary ACR, history of ever smoking and study allocation. In model 2 we additionally adjusted for baseline duration of diabetes, HbA1c, systolic blood pressure, total-cholestrol and HDL-cholesterol, and triglycerides. Since these are factors that BMI can be expected to affect causally, most results presented are from model 1. We also evaluated the association of BMI as a continuous variable with major renal events using piece-wise linear splines with knots at 18.5, 25, 30, 35, 40, and 45 kg/m^2^, and a reference value at 21 kg/m^2^. The hazard ratio for major renal events associated with each single additional unit of BMI above 25 kg/m^2^ was also estimated.

Sensitivity analyses were performed to test the association of BMI categories with the risk of major renal events: (i) in different groups of randomised study treatment (standard and intensive glucose control; placebo and perindopril-indapamide) considered separately; (ii) in different CKD stages (stage 1 [eGFR ≥ 90 mL/min/1.73 m^2^]; stage 2 [≥60 to <90], and stage 3 [<60]); (iii) after treating non-renal death as a competing risk using the Fine and Gray method^18^; (iv) in participants who did not change their BMI category during follow-up; (v) the association of BMI categories with the risk of new microalbuminuria in patients with normoalbuminuria at baseline; and (vi) after including patients with underweight, who were otherwise omitted.

Statistical analyses were performed using SAS software, version 9.3 (SAS Institute, www.sas.com) and Stata software version 13 (StataCorp., www.stata.com). A *p*-value < 0.05 was considered significant.

## Results

### Baseline characteristics by BMI categories

Among 10,537 participants investigated, 58% were men, and 38, 43, and 19% were from Asia, Established market economies, and Eastern Europe, respectively (Table 1). Their mean (SD) age and duration of diabetes were 66(6) and 8(6) years, respectively, and their mean HbA1c was 7.5 (1.5)%. Mean (SD) BMI at baseline was 28(5) kg/m^2^, and 2894 (27%), 4340 (41%), 2265 (22%), 744 (7%) and 294 (3%) patients, respectively, were in the normal weight, overweight and obesity grades 1, 2 and 3 categories. Mean eGFR was 75(17) mL/min/1.73 m^2^, and 2341 (22%), 5952 (57%) and 2244 (21%) were categorised as CKD stages 1, 2, and 3, respectively. Median urinary ACR was 15(7–40) mg/g, with 7312 (69%), 2824 (27%) and 401 (4%) in the normo-albuminuric, micro-albuminuric and macro-albuminuric ranges.

**Table 1:** Characteristics of participants by BMI categories

	Overall (*n* = 10,537)	Normal weight (*n* = 2894)	Overweight (*n* = 4340)	Obesity grade 1 (*n* = 2265)	Obesity grade 2 (*n* = 744)	Obesity grade 3 (*n* = 294)
Male sex, *n* (%)	6063 (57.5)	1658 (57.3)	2687 (61.9)	1255 (55.4)	358 (48.1)	105 (35.7)
Asia, *n* (%)	3988 (37.8)	1998 (69.1)	1661 (38.3)	291 (12.8)	33 (4.4)	5 (1.7)
Established market economies, *n* (%)	4537 (43.1)	681 (23.5)	1896 (43.7)	1279 (56.5)	483 (64.9)	198 (67.3)
Eastern Europe, *n* (%)	2012 (19.1)	215 (7.4)	783 (18.0)	695 (30.7)	228 (30.7)	91 (31.0)
Age (years): mean (SD)	65.8 (6.4)	65.9 (6.3)	66.2 (6.4)	65.8 (6.4)	64.2 (6.2)	63.4 (5.9)
Body mass index (kg/m^2^): mean (SD)	28.3 (5.1)	23.0 (1.5)	27.4 (1.4)	32.0 (1.4)	37.0 (1.4)	44.4 (5.0)
Systolic blood pressure (mmHg): mean (SD)	145 (21)	141 (22)	146 (21)	148 (21)	148 (21)	146 (20)
Diastolic blood pressure (mmHg): mean (SD)	81 (11)	78 (11)	81 (11)	82 (11)	83 (11)	82 (11)
Use of antihypertensive treatment, *n* (%)	7237 (68.9)	1675 (57.9)	3014 (69.5)	1710 (75.5)	590 (79.3)	248 (84.4)
Duration of diabetes (years): mean (SD)	7.9 (6.3)	9.1 (6.9)	7.6 (6.1)	7.4 (6.2)	6.8 (5.8)	7.0 (5.7)
HbA1C (%): mean (SD)	7.5 (1.5)	7.6 (1.8)	7.4 (1.4)	7.5 (1.4)	7.5 (1.4)	7.6 (1.6)
HbA1C (mmol/mol): mean (SD)	58 (17)	60 (19)	57 (16)	58 (16)	59 (16)	60 (17)
eGFR (ml/min/1.73 m^2^): mean (SD)	75 (17)	76 (20)	75 (17)	73 (17)	74 (17)	74 (17)
Urinary ACR (mg/g): median (Q1, Q3)	15 (7, 40)	16 (8, 43)	15 (7, 38)	14 (6, 38)	13 (7, 36)	17 (7, 41)
Serum Total cholesterol (mmol/l): mean (SD)	5.2 (1.2)	5.2 (1.2)	5.2 (1.2)	5.2 (1.2)	5.2 (1.1)	5.3 (1.1)
Serum LDL cholesterol (mmol/l): mean (SD)	3.1 (1.0)	3.1 (1.0)	3.1 (1.0)	3.1 (1.0)	3.1 (1.0)	3.2 (1.1)
Serum HDL cholesterol (mmol/l): mean (SD)	1.3 (0.3)	1.3 (0.4)	1.2 (0.3)	1.2 (0.3)	1.2 (0.3)	1.2 (0.3)
Serum triglycerides (mmol/l)	1.6 (1.2, 2.3)	1.4 (1.0, 2.1)	1.6 (1.2, 2.3)	1.8 (1.3, 2.5)	1.8 (1.4, 2.5)	2.0 (1.4, 2.7)
Use of lipid lowering drugs, *n* (%)	3674 (34.9)	700 (24.2)	1609 (37.1)	910 (40.2)	328 (44.1)	127 (43.2)
History of current smoking, *n* (%)	1579 (15.0)	448 (15.5)	644 (14.8)	307 (13.6)	132 (17.7)	48 (16.3)
History of ever smoking, *n* (%)	4415 (41.9)	941 (32.5)	1859 (42.8)	1080 (47.7)	400 (53.8)	135 (45.9)
Prior cardiovascular disease, *n* (%)	2725 (25.9)	700 (24.2)	1194 (27.5)	571 (25.2)	194 (26.1)	66 (22.5)

Established market economies: Australia, Canada, France, Germany, Ireland, Italy, Netherlands, New Zealand, United Kingdom; Eastern Europe: the Czech Republic, Estonia, Hungary, Lithuania, Poland, Russia, Slovakia; Asia: Philippines, China, Malaysia, India. eGFR, estimated glomerular filtration rate computed by the chronic kidney disease epidemiology collaboration equation. Use of lipid lowering drugs: statins or other hypolipidemic agents. Prior cardiovascular disease: presence at baseline of myocardial infarction, stroke, coronary artery bypass graft, percutaneous transluminal coronary angioplasty, hospital admission for unstable angina or transient ischaemic attack

*ACR* albumin to creatinine ratio

Compared to those with normal weight, patients with obesity were more frequently from established market economies, had a shorter duration of diabetes, and greater systolic blood pressure, and serum triglycerides concentration. They were more likely to use antihypertensive and lipid lowering treatments, and to have ever smoked.

### Risk of major renal events during follow-up by BMI categories

**Fig. 1:**
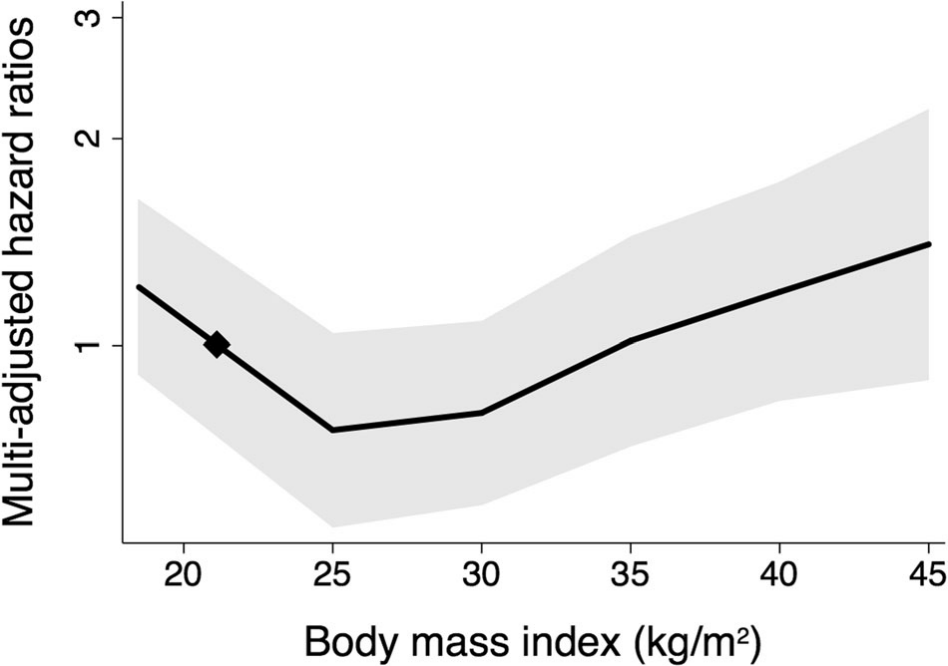
Hazard ratios for a major renal event by BMI splines at baseline Multi-adjusted hazard ratios (solid line) and 95% confidence intervals (shaded region) for major renal events during follow-up according to baseline BMI as a continuous variable with a reference value at 21 kg/m^2^ (diamond). Analyses were adjusted for baseline age, sex, region of origin, prior cardiovascular disease, estimated glomerular filtration rate (and its square), urinary albumin to creatinine ratio, history of ever smoking, and study allocations

Major renal events occurred in 487 (4.6%) participants during a median duration of follow-up of 5.0 (interquartile interval: 4.5–5.0) years. Patients who developed major renal events during follow-up, compared to those who did not, were more frequently men, had a longer duration of diabetes at baseline, higher systolic blood pressure, HbA1c, and urinary ACR levels, had a lower eGFR, and were more likely to use antihypertensive and lipid lowering drugs (Supplemental Table S1). Major renal events occurred in 144 (5.0%), 181 (4.2%), 96 (4.2%), 43 (5.8%), and 23 (7.8%) participants with normal weight, overweight, and obesity grades 1, 2 and 3, respectively (Table 2). The risk of major renal events increased gradually across increasing BMI categories, and the highest risk was observed in patients with severe obesity. Adjusted HRs (95% CIs) from model 1, compared to normal weight, were: overweight: 0.91 (0.72–1.15), obesity grade 1: 1.03 (0.77–1.37), grade 2: 1.42 (0.98–2.07), and grade 3: 2.16 (1.34–3.48, *p* for trend = 0.006). Very similar results were observed when additional adjustments, including mediating factors, were included (model 2)—as was the case for the remaining analyses (results not shown). The same pattern was seen when BMI was fitted as a continuous variable (Fig. 1). Above 25 kg/m^2^, the association of BMI with major renal events appeared to be log-linear, and each additional unit was associated with 4(1–6)% increased risk (*p* = 0.002).

**Table 2:** Major renal events during follow-up according to BMI categories at baseline

	Major renal events (n)		Model 1		Model 2	
	No	Yes	HR (95% CI)	*p* for trend	HR (95% CI)	*p* for trend
Normal weight	2750	144	Ref.	0.006	Ref.	0.01
Overweight	4159	181	0.91 (0.72–1.15)		0.91 (0.72–1.15)	
Obesity grade 1	2169	96	1.03 (0.77–1.37)		1.02 (0.76–1.37)	
Obesity grade 2	701	43	1.42 (0.98–2.07)		1.39 (0.94–2.04)	
Obesity grade 3	271	23	2.16 (1.34–3.48)		2.05 (1.25–3.34)	

Hazard ratios (HR) computed by Cox proportional hazards regression analyses adjusted for baseline age, sex, region of origin, prior cardiovascular disease, estimated glomerular filtration rate (and its square), urinary albumin to creatinine ratio, history of ever smoking, and study allocations (model 1), plus duration of diabetes, HbA1c, systolic blood pressure, total-cholestrol and HDL-cholesterol, and triglycerides (model 2)

### Risk of secondary endpoints during follow-up according to BMI categories at baseline

New cases of microalbuminuria, macroalbuminuria, and doubling of creatinine, ESRD or renal death occurred during follow-up in 2730 (25.9%), 389 (3.5%) and 162 (1.5%) participants, respectively. The risk of new microalbuminuria or macroalbuminuria increased gradually across increasing BMI categories (Table 3). The risk of doubling of creatinine, ESRD or renal death seems to be higher in patients with obesity stages 2 and 3, but the test for trend was non-significant. Each additional unit of BMI over 25 kg/m^2^ increased the risk of microalbuminuria (*p* = 0.0008), macroalbuminuria (*p* = 0.004), and doubling of creatinine, ESRD or renal death (*p* = 0.008) by 2(1–3), 4(1–6), and 5(1–10)%, respectively (using model 1).

**Table 3:** Secondary endpoints according to BMI categories at baseline

	Microalbuminuria (*n*)		Microalbuminuria vs. not		Macroalbuminuria (*n*)		Macroalbuminuria vs. not		Doubling of creatinine, ESRD or renal death (*n*)		Doubling of creatinine, ESRD or renal death vs. not	
	No	Yes	HR	*p* for trend	No	Yes	HR	*p* for trend	No	Yes	HR	*p* for trend
			(95% CI)				(95% CI)				(95% CI)	
Normal weight	2102	792	Ref.	0.01	2779	115	Ref.	0.02	2851	43	Ref.	0.09
Overweight	3229	1111	0.99				0.91				0.84	
			(0.90–1.09)		4198	142	(0.70–1.18)		4290	50	(0.54–1.28)	
Obesity grade 1	1719	546	1.03		2188	77	1.08		2240	25	0.94	
			(0.92–1.17)				(0.78–1.49)				(0.55–1.62)	
Obesity grade 2	536	208	1.28		712	32	1.37		731	13	1.55	
			(1.08–1.51)				(0.89–2.12)				(0.78–3.08)	
Obesity grade 3	221	73	1.19		276	18	2.18		287	7	2.57	
			(0.93–1.53)				(1.27–3.73)				(1.08–6.15)	

Hazard ratios computed by Cox proportional hazards regression analyses adjusted as in model 1: baseline age, sex, region of origin, prior cardiovascular disease, estimated glomerular filtration rate (and its square), urinary albumin to creatinine ratio, history of ever smoking, and study allocations.

### Sensitivity analyses

The associations of BMI categories with the risk of major renal events were compared in different groups of study treatments (Table 4, *p* for interaction between trend in BMI and glucose lowering control = 0.14 and *p* for interaction between trend in BMI and blood pressure treatment = 0.96), as well as in different baseline CKD stages (*p* for interaction = 0.14, Supplemental Table S2) and remained significant after treating non-renal death as a competing risk (*p* for trend = 0.01, Supplemental Table S3). During follow-up, 7103 (67%) participants maintained the same BMI categories as at baseline (Supplemental Table S4). When we considered only these participants, BMI categories remained significantly associated with major renal events (*p* for trend = 0.002, Supplemental Table S5). Similarly, the association of BMI categories with increasing risk of new microalbuminuria remained significant (*p* for trend = 0.02) in patients with normoalbuminuria at baseline (Supplemental Table S6). Finally, when we considered the entire cohort, underweight was associated with a higher risk of major renal events compared to normal weight (HR 2.17, 95% CI 1.01–4.67) (using model 1).

**Table 4:** Major renal events during follow-up according to BMI categories at baseline, in each randomised group

		Major renal events		HR (95% CI)
		No	Yes	
Glucose lowering control (*p* for interaction = 0.14)				
Standard	Normal weight	1374	84	Ref.
	Overweight	2086	100	0.91 (0.67–1.23)
	Obesity grade 1	1063	58	1.08 (0.74–1.57)
	Obesity grade 2	355	16	1.06 (0.60–1.88)
	Obesity grade 3	128	11	2.00 (1.02–3.92)
Intensive	Normal weight	1376	60	Ref.
	Overweight	2073	81	0.90 (0.63–1.28)
	Obesity grade 1	1106	38	0.94 (0.60–1.48)
	Obesity grade 2	346	27	1.72 (1.02–2.91)
	Obesity grade 3	143	12	2.23 (1.13–4.43)
Blood pressure treatment (*p* for interaction = 0.88)				
Placebo	Normal weight	1374	80	Ref.
	Overweight	2072	97	0.86 (0.63–1.17)
	Obesity grade 1	1071	41	0.82 (0.54–1.24)
	Obesity grade 2	369	22	1.36 (0.80–2.30)
	Obesity grade 3	138	15	2.54 (1.37–4.68)
Perindopril-indapamide	Normal weight	1376	64	Ref.
	Overweight	2087	84	0.95 (0.68–1.34)
	Obesity grade 1	1098	55	1.25 (0.84–1.87)
	Obesity grade 2	332	21	1.43 (0.83–2.47)
	Obesity grade 3	133	8	1.61 (0.74–3.52)

Hazard ratios computed by Cox proportional hazards regression analyses adjusted as in model 1: baseline age, sex, region of origin, prior cardiovascular disease, estimated glomerular filtration rate (and its square), urinary albumin to creatinine ratio, history of ever smoking, glucose control (analyses in blood pressure treatment groups) and blood pressure (analyses in glucose control groups) study allocations. The *p*-values represent tests for interaction between study treatment groups

## Discussion

In the current investigation, we evaluated the effect of BMI at baseline on the 5-year risk of major renal events in patients with type 2 diabetes. Above 25 kg/m^2^, the risk of major renal events increased progressively through BMI categories: on average, each 1 unit higher BMI increased this risk by 4%. The increased risk of major renal events was independent of putative risk factors and was observed even after allowing for the competing risk of non-renal death. It was comparable in participants randomly assigned to either standard or intensive glucose control, and to placebo or perindopril-indapamide. Similar associations were also observed when we considered only participants who remained in the same BMI categories during follow-up.

Only a few prospective studies have examined the relationship between BMI and renal events separately among people who have already developed type 2 diabetes, and these have reported a range of findings^9,19,20^. One of the larger studies conducted in 5829 Chinese patients with type 2 diabetes (mean %HbA1 < 8) found an inverse association between BMI and CKD. However, these analyses adjusted for risk factors on the causal pathway between BMI and CKD, including blood pressure, albuminuria, diabetic characteristics, and other traits of metabolic syndrome (e.g. central obesity), and this adjustment may have distorted aetiological associations^20^. Our own larger study of 10,537 patients with type 2 diabetes provides clear evidence for an increased risk of major renal events with increasing BMI over 25 kg/m^2^, in Cox models including BMI both as a categorical and as a continuous variable. The highest risk was observed in patients with morbid obesity.

Despite little apparent cross-sectional association between baseline BMI and baseline urinary ACR in our study, there was a clear positive association between BMI and development of new cases of microalbuminuria and macroalbuminuria, and these hazards were similar in size to the trend toward association between BMI and doubling of creatinine, ESRD or death. Furthermore, each additional unit of BMI over 25 kg/m^2^ increased these endopints by 2, 4, and 5%, respectively. A key mechanism for obesity-associated albuminuria is intraglomerular hypertension, which increases renal blood flow and fractional urinary albumin clearance^21–24^. The consequent mechanical stress results in glomerular enlargement (hypertrophy) and an increased distance between the neighbouring podocytes, damaging a key cellular layer of the glomerular filtration barrier^25^ and perhaps causing podocyte death with focal segmental glomerulosclerosis^26–28^. Randomisation to perindopril + indapamide in ADVANCE reduced total renal events (major renal events plus new microalbuminuria) by 21% (relative risk 0.79, 0.73–0.85)^15^. However, in our subgroup analyses, we found BMI–major renal events associations were not modified by allocated to perindopril + indpamide, which is consistent with a hypothesis that general adiposity may affect renal risk by mechanisms in addition to the haemodynamic stress of glomerular hyperfiltration.

Hyperglycaemia has been suggested as a metabolic podocyte stressor^25^. An inverse association between high insulin sensitivity (estimated by euglycemic clamp) and impaired renal function in a community-based cohort has been reported^29^, and pre-diabetes has been associated with directly measured evidence of hyperfiltration independent of BMI^7^. However, our subgroup analyses suggested that the BMI–major renal events association was not significantly modified by glycaemic control allocation (average HbA1c difference 0.7%), despite the inverse relationship between HbA1c and weight^30^. Another mechanism by which adipose tissue may cause kidney disease is the visceral fat deposition in the renal sinus, which may compress the main renal artery and vein^31–33^, but measurements relevant to these mechanisms were not measured in this study.

Nevertheless, our findings are consistent with reports that weight loss may protect against the development of renal complications in overweight or obese individuals with type 2 diabetes. The Look AHEAD (Action for Health in Diabetes) trial showed that intensive lifestyle intervention, compared to standard education, resulted in 8% weight loss (on average 4 kg) and a consequent 31% reduction (hazard ratio 0.69 [0.55–0.87]) in “very-high-risk CKD” (based in KDIGO risk charts)^34^. Weight loss may also be one of the mechanisms by which sodium-glucose co-transporter 2 inhibitors or analogues of glucagon-like peptide 1 reduce renal risk^35–37^. Lastly, bariatric surgery has been associated with an improvement in renal function^38–40^. Regardless of the mechanism, higher risk with obesity suggests there may be greater absolute benefit from attention to all risk factors among patients with obesity and diabetes at risk for CKD progression.

The present investigation’s key strength was its comprehensive clinical and biological characterisation of participants, and 5 years of prospective follow-up including pre-specified renal outcomes confirmed by an independent adjudication committee^13^. However, it is possible that the number of major renal events may have been insufficiently large to identify important differences between the randomised groups. Also, ADVANCE did not collect detailed data on body fat distribution, so that important differences between body-mass composition between participants could not be assessed for its relevance to major renal events. Furthermore, creatinine determinations were not isotope dilution mass spectrometry (IDMS) traceable in the ADVANCE trial as all participants were enroled before the international recommendations for IDMS alignment^41,42^.

In conclusion, obesity at different stages was an independent predictor of major renal events in patients with type 2 diabetes. Our findings encourage comprehensive and motivated weight loss programmes for improving the prevention of the development and progression of kidney complications in patients with both type 2 diabetes and obesity.

## Electronic supplementary material

SUPPLEMENTAL TABLES
