# Supplementary material for: Associations between body mass index and the risk of renal events in patients with type 2 diabetes
Source: Nutr Diabetes. 2018 Jan 17;8:7. doi: 10.1038/s41387-017-0012-y (PMC5851426; doi:10.1038/s41387-017-0012-y)
Supplement: Supplementary file 1 — SUPPLEMENTAL TABLES [file 41387_2017_12_MOESM1_ESM.docx]

**Supplemental Table S1. Characteristics of participants at baseline according to the incidence of major renal events during follow-up**

|  | Major renal events | |
| --- | --- | --- |
|  | No  (n=10050) | Yes  (n=487) |
| Male sex, n (%) | 5738 (57.1) | 325 (66.7) |
| Asia, n (%) | 3779 (37.6) | 209 (42.9) |
| Established market economies, n (%) | 4324 (43.0) | 213 (43.7) |
| Eastern Europe, n (%) | 1947 (19.4) | 65 (13.4) |
| Age (years): mean (SD) | 65.8 (6.4) | 65.9 (6.5) |
| Body mass index (kg/m^2^): mean (SD) | 28.3 (5.1) | 28.6 (5.8) |
| Systolic blood pressure (mmHg): mean (SD) | 145 (21) | 151 (22) |
| Diastolic blood pressure (mmHg): mean (SD) | 81 (11) | 81 (12) |
| Use of antihypertensive treatment, n (%) | 6853 (68.2) | 384 (78.9) |
| Duration of diabetes (years): mean (SD) | 7.8 (6.3) | 9.4 (6.7) |
| HbA1c (%): mean (SD) | 7.5 (1.5) | 8.0 (1.8) |
| HbA1c (mmol/mol): mean (SD) | 58 (17) | 64 (20) |
| eGFR (ml/min/1.73 m^2^): mean (SD) | 75 (17) | 66 (20) |
| Urinary ACR (mg/g): median (Q1, Q3) | 14 (7, 34) | 78 (29, 165) |
| Serum Total cholesterol (mmol/l): mean (SD) | 5.2 (1.2) | 5.1 (1.2) |
| Serum LDL cholesterol (mmol/l): mean (SD) | 3.1 (1.0) | 3.1 (1.1) |
| Serum HDL cholesterol (mmol/l): mean (SD) | 1.3 (0.3) | 1.2 (0.4) |
| Serum triglycerides (mmol/l) : median (Q1, Q3) | 1.6 (1.2, 2.3) | 1.8 (1.3, 2.6) |
| Use of lipid lowering drugs, n (%) | 3474 (34.6) | 200 (41.1) |
| History of current smoking, n (%) | 1497 (14.9) | 82 (16.8) |
| History of ever smoking, n (%) | 4186 (41.7) | 229 (47.0) |
| Prior cardiovascular disease, n (%) | 2572 (25.6) | 153 (31.4) |

Established market economies: Australia, Canada, France, Germany, Ireland, Italy, Netherlands, New Zealand, United Kingdom; Eastern Europe: the Czech Republic, Estonia, Hungary, Lithuania, Poland, Russia, Slovakia; Asia: Philippines, China, Malaysia, India. eGFR, estimated Glomerular Filtration Rate computed by the Chronic Kidney Disease Epidemiology Collaboration equation. ACR, Albumin to Creatinine Ratio. Use of lipid lowering drugs: statins or other hypolipidemic agents. Prior cardiovascular disease: presence at baseline of myocardial infarction, stroke, coronary artery bypass graft, percutaneous transluminal coronary angioplasty, hospital admission for unstable angina or transient ischaemic attack.

**Supplemental Table S2 . Major renal events during follow-up according to BMI categories at baseline by chronic kidney disease stages**

|  |  | Major renal events (n) | |  |
| --- | --- | --- | --- | --- |
|  |  | No | Yes | HR (95% CI) |
| Chronic kidney disease stages (p for interaction = 0.14) | | |  |  |
| CKD stage 1 | Normal weight | 729 | 24 | Ref. |
|  | Overweight | 915 | 20 | 0.61 (0.33 – 1.13) |
|  | Obesity grade 1 | 411 | 19 | 1.19 (0.59 – 2.39) |
|  | Obesity grade 2 | 152 | 7 | 1.03 (0.39 – 2.72) |
|  | Obesity grade 3 | 59 | 5 | 2.62 (0.88 – 7.79) |
| CKD stage 2 | Normal weight | 1520 | 70 | Ref. |
|  | Overweight | 2398 | 83 | 0.84 (0.60 – 1.17) |
|  | Obesity grade 1 | 1261 | 43 | 0.92 (0.60 – 1.41) |
|  | Obesity grade 2 | 392 | 20 | 1.67 (0.96 – 2.89) |
|  | Obesity grade 3 | 154 | 11 | 2.46 (1.24 – 4.88) |
| CKD stage 3 | Normal weight | 501 | 50 | Ref. |
|  | Overweight | 846 | 78 | 1.14 (0.78 – 1.65) |
|  | Obesity grade 1 | 497 | 34 | 1.02 (0.63 – 1.65) |
|  | Obesity grade 2 | 157 | 16 | 1.21 (0.65 – 2.27) |
|  | Obesity grade 3 | 58 | 7 | 1.68 (0.72 – 3.92) |

Hazard ratios (HR) computed by Cox proportional hazards regression analyses adjusted for baseline age, sex, region of origin, prior cardiovascular disease, urinary albumin to creatinine ratio, history of ever smoking, and study allocations. Chronic kidney disease (CKD) stage 1 (eGFR ≥90 mL/min/1.73m^2^), satge 2 (≥60 – <90) and stage 3 (<60).

**Supplemental Table S3. Major renal events during follow-up according to BMI categories at baseline with correction for competing risk of non-renal death**

|  |  | Major renal events *vs.* not | |
| --- | --- | --- | --- |
|  |  | Subhazard ratio (95% CI) | P for trend |
| Overweight *vs.* normal weight |  | 0.91 (0.72 – 1.14) | 0.01 |
| Obesity grade 1 *vs.* normal weight |  | 1.03 (0.78 – 1.37) |  |
| Obesity grade 2 *vs.* normal weight |  | 1.40 (0.96 – 2.03) |  |
| Obesity grade 3 *vs.* normal weight |  | 2.12 (1.31 – 3.43) |  |

Subdistribution hazard ratios adjusted as in model 1: baseline age, sex, region of origin, prior cardiovascular disease, estimated glomerular filtration rate (and its square), urinary albumin to creatinine ratio, history of ever smoking, and study allocations.

**Supplemental Table S4. Changes in BMI categories during follow-up**

|  | BMI categories during follow-up | | | | | |
| --- | --- | --- | --- | --- | --- | --- |
|  | Underweight | Normal weight | Overweight | Obesity grade 1 | Obesity grade 2 | Obesity grade 3 |
| BMI categories  at baseline |  |  |  |  |  |  |
| Normal weight | 50 (1.8) | **2219 (78.1)** | 541 (19.0) | 26 (0.9) | 3 (0.1) | 1 (0.1) |
| Overweight | 7 (0.2) | 754 (17.8) | **2949 (69.6)** | 509 (12.0) | 14 (0.3) | 1 (0.1) |
| Obesity grade 1 | 0 | 30 (1.4) | 520 (23.6) | **1352 (61.4)** | 287 (13.0) | 14 (0.6) |
| Obesity grade 2 | 0 | 3 (0.4) | 23 (3.2) | 201 (27.6) | **394 (54.2)** | 106 (14.6) |
| Obesity grade 3 | 0 | 6 (2.1) | 2 (0.7) | 17 (5.9) | 73 (25.4) | **189 (65.9)** |

Data expressed as the number of patients (corresponding percentage). Stable categories are shown in bold.

**Supplemental Table S5. Major renal events during follow-up according to BMI categories at baseline in participants with stable BMI categories during follow-up (n=7103)**

|  | Major renal events (n) | |  | |
| --- | --- | --- | --- | --- |
|  | No | Yes | HR (95% CI) | P for trend |
| Normal weight | 2113 | 106 | Ref. | 0.002 |
| Overweight | 2832 | 117 | 0.90 (0.68 – 1.19) |  |
| Obesity grade 1 | 1294 | 58 | 1.07 (0.74 - 1.53) |  |
| Obesity grade 2 | 366 | 28 | 2.07 (1.30 – 3.30) |  |
| Obesity grade 3 | 174 | 15 | 2.37 (1.31 – 4.28) |  |

Hazard ratios (HR) computed by Cox proportional hazards regression analyses adjusted as in model 1: baseline age, sex, region of origin, prior cardiovascular disease, estimated glomerular filtration rate (and its square), urinary albumin to creatinine ratio, history of ever smoking, and study allocations.

**Supplemental Table S6. Risk of new cases of microalbuminuria during follow-up in normoalbuminuric patients at baseline**

|  | Microalbuminuria (n) | | Microalbuminuria *vs.* not | |
| --- | --- | --- | --- | --- |
|  | No | Yes | HR (95% CI) | P for trend |
| Normal weight | 1172 | 775 | Ref. | 0.02 |
| Overweight | 1962 | 1084 | 0.98 (0.89 – 1.08) |  |
| Obesity grade 1 | 1064 | 538 | 1.03 (0.91 - 1.16) |  |
| Obesity grade 2 | 322 | 203 | 1.26 (1.07 - 1.49) |  |
| Obesity grade 3 | 123 | 69 | 1.19 (0.92 - 1.54) |  |

Hazard ratios computed by Cox proportional hazards regression analyses adjusted as in model 1: baseline age, sex, region of origin, prior cardiovascular disease, estimated glomerular filtration rate (and its square), urinary albumin to creatinine ratio, history of ever smoking, and study allocations.
